# Nano-magnolol enhances the modulatory effects of magnolol on cognitive performance and BACE1-related biochemical changes in an STZ-induced rat model of Alzheimer’s disease

**DOI:** 10.1186/s11671-026-04506-9

**Published:** 2026-04-09

**Authors:** Basma Youssef, Ehab A. Ibrahim, Said S. Moselhy, Shaimaa ElShebiney, Walaa K. ELabd

**Affiliations:** 1 Biochemistry Department, Faculty of Science, Ain Shams University, Cairo, Egypt; 2 Department of Narcotics, Ergogenics and Poisons National Research Centre, Cairo, Egypt

**Keywords:** Magnolol nanoparticles, β-secretase1, Nuclear factor kappa B, Alzheimer, Rats

## Abstract

**Background:**

The Late-onset Alzheimer’s disease (LOAD) is progressive cognitive deficits associated with different abnormalities as cholinergic dysfunction, amyloid accumulation, inflammation, and oxidative stress. Magnolol is a polyphenolic compound that abrogated the neurodegenerative disease. The application of nanoparticles in medicine showed high bioavailability and low side effects for development of novel effective therapies. This study evaluated the neuroprotective potential of magnolol nanoparticles against streptozotocin (STZ) injected in intracerebroventricularly (ICV) induced Alzheimer’s disease (AD) in rats.

**Methods:**

In current study, six groups of male Wister rats (10 rats/ group) were injected with STZ (2 mg/kg) in ICV bilaterally for induction of pathological features similar to AD. Rats were then treated with either magnolol or nano-magnolol or donepezil (*p.o*). Behavioral analysis was evaluated as the Morris Water Maze (MWM), Y-Maze, Novel Object Recognition (NOR), Passive Avoidance (PA), Elevated plus Maze (EPM), and Open Field Test (OFT). In addition, biochemical markers including brain acetylcholinesterase (AChE), glutathione-S-transferase (GST), B-secretase1 (BACE1) activities and nuclear factor kappa-B (NF-κB) were analyzed in hippocampal tissue.

**Results:**

Data obtained showed that nano-magnolol significantly showed a neuroprotective effect in LOAD rat model by restoring GST activity and effectively decreased the activities of AChE, BACE1 and level of NF-κB compared to both donepezil and magnolol. Molecular docking studies indicated strengthen the affinity of magnolol to the BACE-1 active site.

**Conclusion:**

Nano-magnolol is promising in developing a new agent targeting cholinergic function, amyloidogenesis, neuro-inflammation, and oxidative stress reflecting its potent neuroprotective efficacy in AD treatment.

**Supplementary Information:**

The online version contains supplementary material available at 10.1186/s11671-026-04506-9.

## Introduction

Alzheimer’s disease (AD) is a multifactorial neurodegenerative disease caused by complicated interactions between lifestyle, metabolic, vascular, and genetic factors. Physical inactivity, poor diet, long-term stress, smoking, and sleep disruptions are examples of lifestyle behaviors that significantly impact cognitive resilience through epigenetic mechanisms, including DNA methylation [45, 86]. As demonstrated by metabolic collapse models, insulin resistance, type 2 diabetes, and hyperglycemia all trigger oxidative stress and mitochondrial dysfunction, which are early stages in the pathophysiology of AD. Cardiovascular and metabolic comorbidities, such as dyslipidemia and hypertension, can significantly increase the risk of dementia [35, 72]. The most serious genetic risk factor for LOAD has been shown to be the apolipoprotein E (APOE-ε4) allele, which disrupts lipid metabolism in the brain and dramatically increases Aβ accumulation in various stages of life [10].

The prevalence of neurodegenerative diseases has grown in parallel with improvements in living standards because these advancements have led to increased life expectancy, making the population older [18, 74]. The AD is the most prevalent form of dementia, a progressive neurological disorder affecting the elderly [33, 47]. Patients diagnosed with AD suffering from gradual deterioration of memory and cognitive function, accompanied by behavioral disturbances and neuropsychiatric symptoms [7, 62]. It was reported that, the shrinkage of hippocampal and cerebral cortical areas is related to AD, especially in the frontal–temporal horn, resulting in memory and spatial learning impairments [40, 42]. There are approximately 50 million dementia sufferers worldwide, this will be expected to reach 139 million by 2050. The main hallmarks of AD are β-amyloid peptide (Aβ) senile plaques and hyperphosphorylated tau protein neurofibrillary tangles (NFT) [23]. There are two forms of AD, Early-onset AD (EOAD is not related to amyloid burden, has a more aggressive cognitive deterioration than LOAD [53].

There is no curative treatment for AD, currently FDA-approved medications for mitigate the symptomes [8]. For the management of memory impairment, AChE inhibitors are widely utilized, since it is postulated that reducing acetylcholine levels or elevating AChE activity is linked to cognitive impairment [16, 77].

The neuroprotective effects of several active compounds against AD have been studied. Available pharmaceuticals agents such as Tacrine (Cognex), Donepezil (Aricept), and Galantamine (Razadyne) are commonly prescribed as acetylcholinesterase inhibitor, improve cholinergic neurotransmission and cognitive improvement [31, 49]. Concurrently, natural polyphenols as resveratrol and curcumin have drawn interest due to their anti-inflammatory, anti-amyloid, and antioxidant properties [55, 64].

Magnolol is one of Magnolia Officinalis’ key active components [24] that showed different biological activities including cardiovascular protection [52], anti-angiogenesis [79], hypoglycemic [17, 71], gastrointestinal protection [41], antibacterial [70], anti-cancer [41], anti-inflammatory [28], anti-oxidant [13, 30] and [54], and has neuroprotective properties [68]. Moreover, it reduces oxidative stress and neuro-inflammation in the prefrontal cortex of mice [15] and in a rat model induced by formalin without affecting cognitive or locomotor abilities [2].

Several tiny nano carriers, have been used to treat neurological disorders like AD and brain tumors [11]. These nano-carriers are categorized as nanomedicines since they carry particular medications. By reducing dosage and frequency, nanomedicines could boost patient compliance [4]. Aside from clinical concerns, nanomedicines may be superior to other traditional drug administration methods, particularly for the brain, in terms of targeting, stability, biocompatibility, protection against enzymatic drug degradation, longer half-life, enhanced bioavailability, and biodegradability [4, 82]. Nanomedicine promises novel method to treat AD by facilitating the transfer of therapeutic agents across the blood–brain barrier [19, 32]. These nano-carriers include polymeric nanoparticles, nano-suspension, liposomes, and solid lipid nanoparticles [29]. Nano-suspensions are submicron colloidal dispersions made up of pure drug nanoparticles stabilized by surfactants or polymers. As a result, nano-suspensions provide a diverse and efficient platform for improving drug delivery performance and therapeutic efficacy [58]. This study investigated the potential impact of nano-magnolol in cognitive performance and BACE1-related biochemical changes in LOAD induced in rats and its mechanism of action.

## Materials and methods

### Materials

Magnolol (Nutricrafters, Sparks, NV, USA) was obtained from a local pharmacy as a dietary supplement and dispersed in distilled water to be administered orally. Donepezil hydrochloride and streptozotocin were obtained from Sigma Aldrich (St. Louis, MO, USA).

### Preparation of magnolol nanoparticle

Nano-suspension of magnolol was prepared by a method of a bottom-up/top-down approach according to [81]. Briefly, magnolol (500 mg) was dissolved in 6 mL of dimethyl sulfoxide. The solution was then placed into a 0.5% aqueous polyvinyl alcohol (PVA) surfactant solution (50 mL), which was ultrasonicated at 40 kHz (probe sonicator, Hielscher Ultrasonics GmbH, Teltow, Germany( for 2 min before being homogenized using a high-pressure homogenizer (Stansted SPCH-10; Stansted Fluid Power Ltd, Harlow, UK ( for 5 cycles at 1,000 bar. The nano-suspension was centrifuged at 11,000 rpm for 90 min at 4 °C. The obtained nanoparticles are then rinsed with distilled water, centrifuged again under the same conditions, and lyophilized [22].

### Animals

Male Wistar rats weighing between 240 and 300 g, 4 months of age were purchased from the National Research Center breeding colony, Cairo, Egypt. Rats were housed in typical environmental circumstances with a regular light cycle and 24–26 ^ο^C. A standard chow diet and unlimited access to tap water were given, with the exception of the trial period.

### Transmission electron microscopy (TEM)

The morphology and particle size of prepared nanomagnolol were examined using transmission electron microscopy (TEM; JEOL, Japan, JEM2100, ELECTRON MICROSCPE, TEM-HR). The sample was dispersed in ethanol and sonicated for 15 min using an ultrasonic bath to ensure uniform dispersion. A copper grid was then immersed in the sonicated suspension, air-dried and analyzed using TEM to assess the nanoscale features.

### Induction of LOAD model in rats

A subdiabetogenic dosage (2 mg/kg) of STZ was injected intracerebroventricularly bilaterally according to [48] to produce pathological features of AD [37, 60]. The rats were subjected to general anesthesia with Ketamine/ Xylazine cocktail (80/8 mg/kg, i.p) [63]. The skullcap was drilled carefully up to the level of the dura mater. A preventive antibiotic ciprofloxacin (5 mg/kg, i.p.) was used along with NSAID analgesic (ketoprofen 5 mg/kg, s.c.). A sterile needle was slowly lowered into the lateral ventricle using the standard stereotaxic coordinates (AP-0.8 mm, ML ± 1.4 mm, and DV 3.6 mm). 2 µL of STZ was infused through a micropump at 1 µL/min rate in each ventricle equivalent to 2 mg/kg [67]. Thereafter, the needle was held in place for 2 more minutes to prevent reflux and gently withdrawn and the skin was sutured. Sham rats (subjected to the same surgical operation steps) received saline instead of STZ applying the same procedure. Rats were allowed to recover for 3 days after surgery.

### Experimental design

**Fig. 1:**
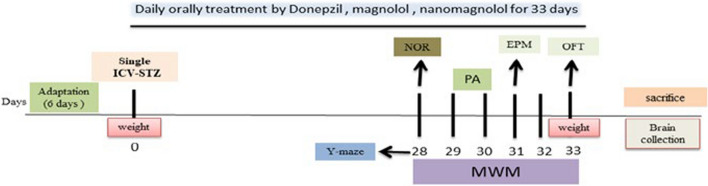
Diagrammatic sketch showing experimental protocol. ICV-STZ: intracerebroventricular-streptozotocin; NOR: novel object recognition; PA: passive avoidance; MWM: Morris water maze; OFT: open field test; EPM: elevated plus maze sacrificed after behavioral assessment for further experiments. Day 0 refers to the day of stereotaxic surgery (ICV injection of STZ). Experimental rats were treated with different treatments for 33 days

Rats were randomly divided into six groups (n = 10). The different treatments were applied from 3rd day till day 33, as shown in Fig. 1. The treatments included saline (1 ml, p.o.) for normal, sham and STZ control animals, **Donepzil** (2.5 mg/kg/day, p.o.) [1], **Magnolol** (20 mg/kg/day, p.o.) [78], and **nanomagnolol** (20 mg/kg/day, p.o.,) [14, 21].

Rats were weighed at the start and end of experiment. Behavioral studies were conducted. Following the animals’ euthanasia, brain tissues were collected, and hippocampus samples were taken out and kept at − 80 °C for further examination.

### Behavioral investigations

According to ARRIVE guidelines 24 h were allowed between tests to ensure sufficient rest periods, reliable and unbiased behavioral outcomes.

#### Passive avoidance test (PA)

This test was used for measuring avoidance memory retention in rodents. Rats were habituated to a two-compartment chamber with the separating door open, after habituation, rats were placed in the lighted compartment for an acquisition trial. After 30 s, the door was raised, and an electrical shock (0.5 mA) was delivered for 5 s when the rat moved to the dark area. After 24 h, the same procedure was repeated but without electric shock. In both the acquisition and retention trials, the transfer latency time (sec) for each rat was recorded [5].

#### Morris water maze (MWM) test

The experimental procedure was described in the previously published Morris water maze test method [75]. The water maze is a circular tank with a diameter of 100 cm and a height of 60 cm with a movable platform (10 × 10 cm). Four starting points at each direction (E, W, N, and S) were equally distributed around the edges, and the water maze was imaginary divided into four quadrants, the platform was set the second quadrant (W). The water immersed the platform by 0.5 cm above its surface, and the water temperature was controlled at about 24 ± 1 °C. One week before the test, 4 platform learning trials were conducted. Each rat was placed in the water from each direction sequentially, and left till climbed up to the platform on all fours within 60 sec. If failed, it was guided gently to the platform and left to rest and learn for 20 sec. escape latency was recorded. On the test day, the platform was taken out, and the each rat was subjected to a 60 sec spatial probe test. To assess the spatial memory power, the amount of time spent in the platform quadrant and the number of quadrant crossings were noted.

#### Y-maze spontaneous alternation test

The Y-maze behavioral assessment was performed as previously described [38]. The test was conducted using a Y-shaped maze with three white opaque arms at an angle of 120° from each other. Rats were introduced to the central location in the maze and allowed to explore freely for 10 min. Alternation was defined as entering all three arms consecutively. The number of arm alternations was monitored. The spontaneous conversion rate was calculated from the equation: The spontaneous conversion rate was calculated = total number of alternations/maximum number of alternations* 100%.

#### Novel object recognition test (NOR)

Cognitive function was additionally assessed by novel object recognition test. A rat was presented with two similar objects (A) during the first 15 min session, and then one of the two objects was replaced by a new object (B) during a second 15 min session 24 h later. The amount of time taken to explore the new object provides an index of recognition memory [44]. The used objects were wooden colored shapes, heavy enough not to be displaced by the animals (~ 15 cm high). The exploration calculated time included sniffing, licking, or touching the object, but not standing or leaning against the object. The discrimination index (DI) was calculated according to the formula: DI = (EB − EA)/(EA + EB) [2].

#### Elevated plus maze test (EPM)

Animals were assessed for anxiety-like behavior using the EPM test. Rats were tested in the two-arms plus maze for five minutes, and each rat was set free in the central area. The overall amount of time spent in the closed arms, the number of entries, and the initial entry time all were recorded [3]. After each rat, 70% ethanol was used to clean the arms.

#### Open field test

Within five minutes, the evaluation was completed in a 100 × 100 cm white-walled box. Following the release of each rat in the middle of the field, numbers of crossings (the number of squares traversed in a manner resembling horizontal activity), crossings to central zones, and grooming time (the amount of time the animal spent grooming its face, licking/cleaning, and scratching different parts of its body) were recorded [76]. After each session, the arena was wiped with a 70% ethanol solution.

### Biochemical investigations

#### Preparation of brain tissue homogenate

Hippocampus tissues were separated from all rats, hemolyzed blood was removed to avoid interference with the result using ice-cold phosphate buffer saline PBS (0.01 M, pH = 7.4). Tissue was weighed and homogenized in PBS (1:9) with a glass homogenizer on ice, sonicated, and centrifuged for 5–10 min at 5000 × *g* at 2–8 °C to obtain supernatant (Cat No. E-EL-R0673).

#### Determination of brain tissue AchE activity, GST, BACE1, and NF-kB

The AChE activity was assessed according to the method of Ellman et al. [20]. For GST, a commercial kit was utilized (Bio Diagnostic, Cat. No. GR 25 25, Egypt). ELISA commercial kits for the Rat NF-κB p105 Kit (Cat No. E-EL-R0673), and BACE1 (Cat No. MBS2512086) were used.

#### In-silico molecular docking study on BACE1

AutoDock Vina modeling simulation software (AutoDock Vina v.1.5.6) was used to predict the protein–ligand binding affinity, as well as the preferred orientation of the docking pose between the amino acid residues that form the active site of the BACE1 (PDB: 6OD6) and magnolol, in addition to the co-crystalized ligand Pyrrolopiperazine Inhibitor; N-{3-[(3R)-1-amino-3-methyl-3,4-dihydropyrrolo[1,2-a]pyrazin-3-yl]-4-fluorophenyl}-5-cyanopyridine-2 carboxamide (PDB: M7D), which was used as a reference ligand as shown in supplementary Figs. S1 and S2.

PyMOL molecular visualization tool (PyMOL v.3.0.5) was used to extract the BACE1protein from its co-crystallized ligand. The extracted files were in PDB format. Auto-Dock (MGL-tools) was used to determine the grid box dimensions that define the three dimensional space for the docking site and the favorable place for binding between the protein and ligand. The grid box dimensions were selected by centering the grid box on M7D and identifying the central points including center X (− 36.007), center Y (− 47.694), and center Z (19.877) and also the grid box size X (52), size Y (40), and size Z (40). Moreover, the target protein and the tested ligand were exported in PDBQT format (AutoDock format) using Open Babel v.2.4.1.

Nine poses were considered for each molecule, with the target protein serving as the rigid receptor and the ligands’ conformation kept as flexible. Finally, the most favorable pose was selected based on the minimum free energy of the protein–ligand complex. For visualizing the type of interactions between the ligand and the protein, BIOVIA Discovery Studio (DS) Visualizer v.24.1.0 was used.

In the present study, docking of magnolol and co-crystallized reference ligand with BACE1 protein (PDB: 6OD6) was performed using AutoDock vina modeling simulation software, by placing the small molecules into the binding site of the target to preliminarily predict the protein-binding affinity, as well as the preferred orientation of the docking pose.

### Statistical analysis

The results of all experiments are shown as mean ± SEM. GraphPad Prism version 8.0 (CA, USA) was used for the analysis, and significant differences were assessed using one-way ANOVA followed by Tukey’s post-hoc test (significant at *p* < 0.05, very significant *p* < 0.01, and highly significant *p* < 0.001).

## Results

### Nano magnolol by transmission electron microscope

#### Particle size

**Fig. 2:**
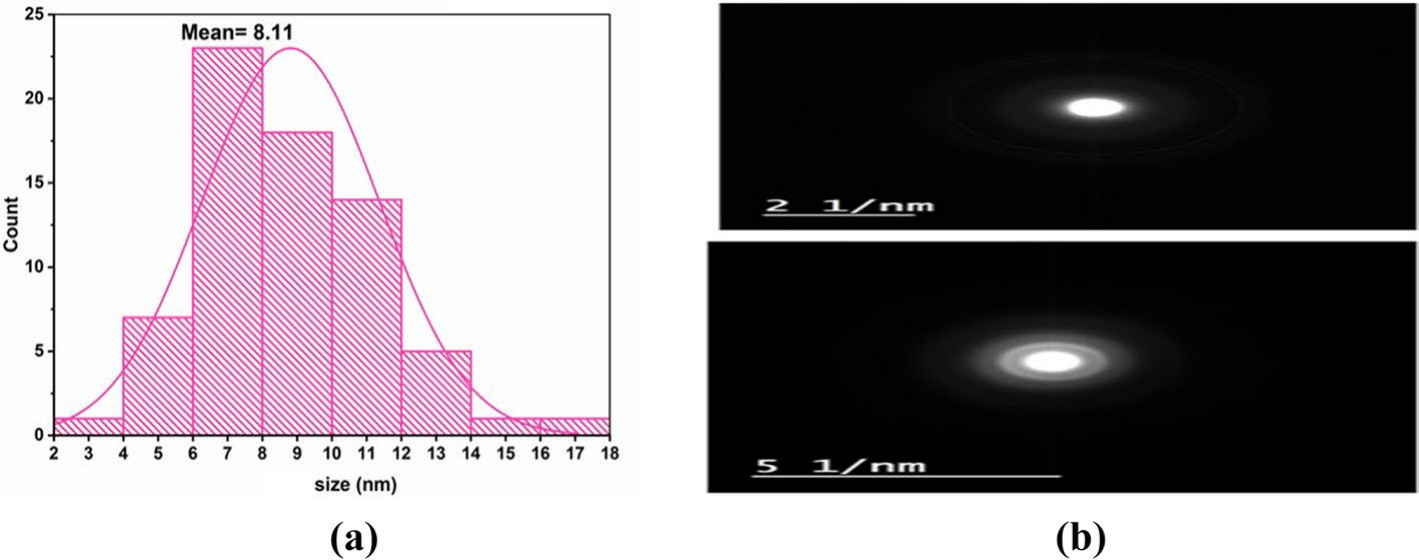
**a** histogram showing the average diameter size of magnolol nanoparticles and **b** Crystalline shape of magnolol nanoparticles

Data shown in Fig. 2A indicated that, magnolol nanoparticle size distribution histogram as determined by TEM and analyzed by Image J software, with an average diameter of 8.11 nm.

#### Morphology

**Fig. 3:**
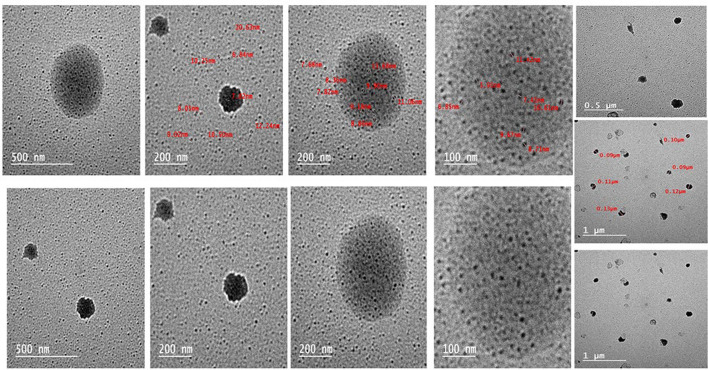
Spherical shapes of magnolol nanoparticles under a transmission electron microscope (TEM)

The spherical shape of the Nano magnolol particles, which ranged in size from 5.9 to 13.68 nm, was verified by TEM pictures as shown in Fig. 3. There was no apparent aggregation and the particles seemed evenly distributed. The crystalline structure of the nanoparticles was demonstrated by the clear diffraction rings seen in Selected Area Electron Diffraction (SAED) patterns as shown in Fig. 2B. These results validate the effective synthesis of crystalline, homogeneous, and stable nanoparticles appropriate for additional biological uses.

### Changes in body weight

**Fig. 4:**
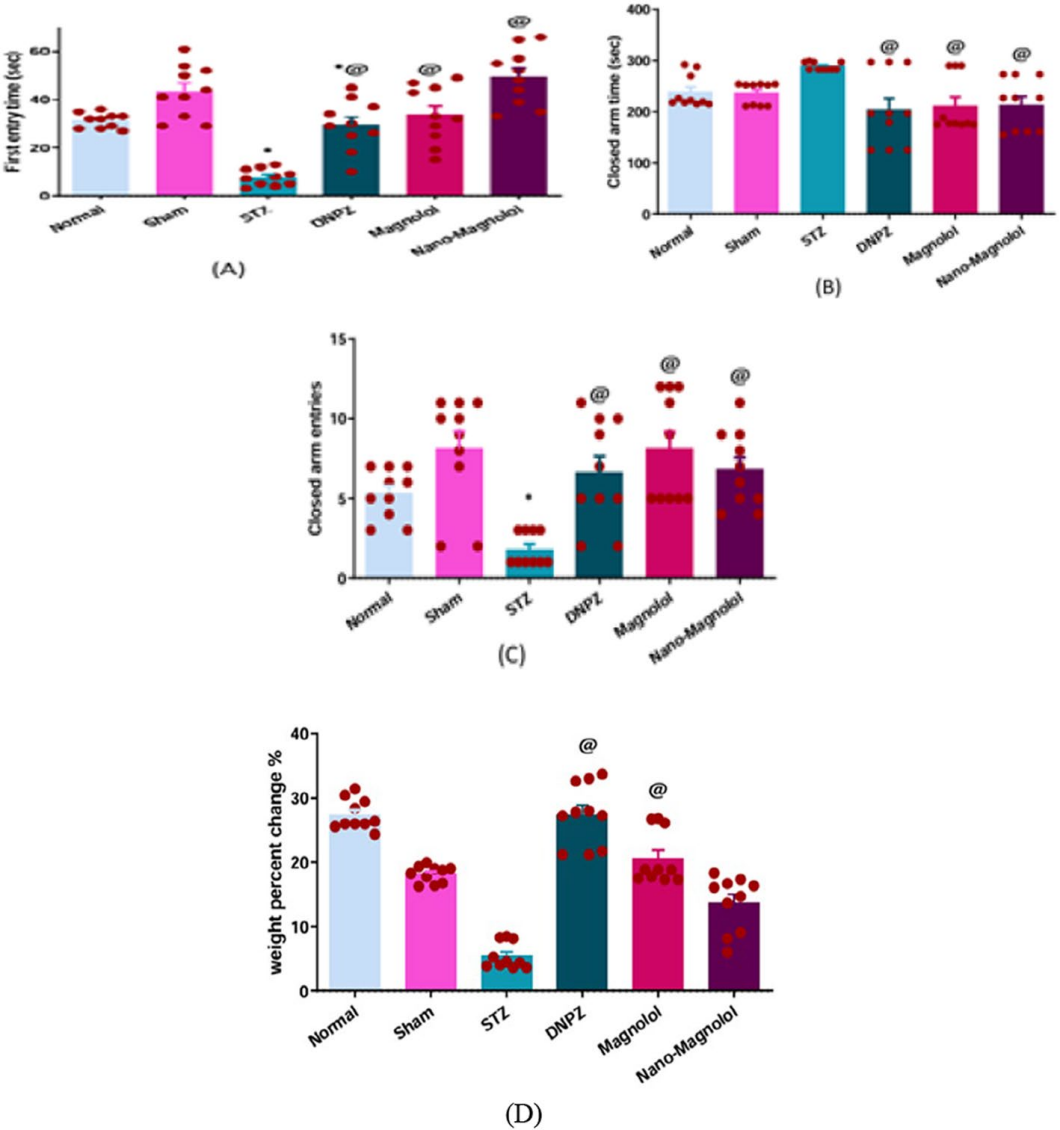
Elevated Plus Maze **A** First entry time **B** time spent in the closed arm and **C** a number of entries in all Elevated Plus Maze experimental groups, and **D** Body weight percent change of the rats in all experimental groups. *Represents a significant difference *p* < 0.001 from the sham control group and @ represents a significant difference *p* < 0.001 from the STZ group after 33 days of the experiment (significant at *p* < 0.05, very significant *p* < 0.01, and highly significant *p* < 0.001)

During the basal week (before the LOAD model), there were no significant changes in the initial body weight between all groups. After four weeks of the experiment, the percent change in body weight varied notably among the experimental groups. While the normal and sham control groups showed noticeable changes, In comparison to their weight in a baseline week, normal control and sham rats gained 27.39 and 18.14% of their initial weight, respectively, the LOAD-induced group exhibited a marginal weight gain of 5.3%. Interestingly, animals receiving treatments of donepzil, magnolol and nano-magnolol also demonstrated a significant increase (*p* < 0.001) in body weight compared to the LOAD control group Fig. 4D.

### Elevated plus maze (EPM)

In the EPM test, behavioral differences were observed across the groups in terms of anxiety-related parameters. The LOAD group exhibited a marked anxiety-like behavior, as indicated by a shorter latency to first entry into the closed arm (*p* < 0.001) compared to sham control animals (7.70 ± 1.10 s vs 43.40 ± 3.45 s), suggesting a faster withdrawal response. Furthermore, compared to sham control animals, this group spent much more time in the closed arms (*p* < 0.001). (288.80 ± 2.37 s vs 236.4 ± 6.78 s) and a lower number of entries into the closed arms compared to sham control animals (1.80 ± 0.32 vs 8.10 ± 1.10, *p* < 0.001), consistent with increased anxiety levels. On the other hand, Donepzil-treated, magnolol-treated and nano-magnolol treated groups demonstrated delayed first entry into the closed arms (29.50 ± 3.32 s, 33.70 ± 3.78 s and 49.40 ± 3.69 s) respectively in comparison to the LOAD group Fig. 4A, less time spent in the closed arms (203.60 ± 22.47 s, 211.40 ± 17.19 s, and 213.80 ± 15.94 s) respectively in comparison to the LOAD group Fig. 4B, and significantly higher entries (6.60 ± 1.04, (8.10 ± 1.07, and 6.80 ± 0.75, *p* < 0.001) respectively in comparison to the LOAD group Fig. 4C, suggesting reduced anxiety-like behavior and improved exploratory responses. The normal control and sham groups exhibited typical exploratory patterns, with moderate latency to closed arm entry and balanced time distribution between arms.

### Open field test

**Fig. 5:**
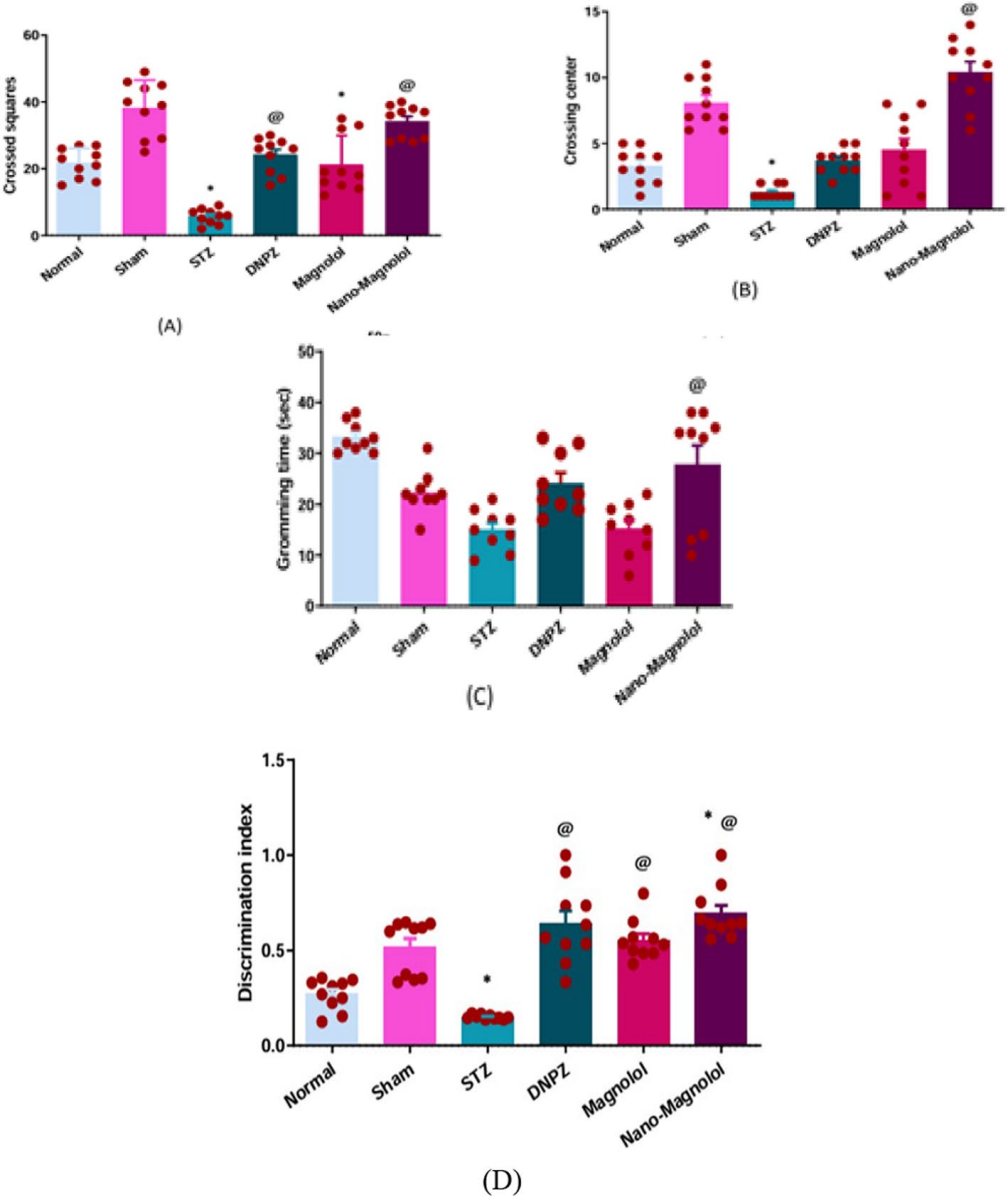
Open Field Test (OFT) **A** crossed squares **B** crossing center and **C** grooming time in all OFT, and **D** Novel object recognition test (DI) of all experimental groups. *Represents a significant difference *p* < 0.001 from the sham control group after 33 days of the experiment and @ represents a significant difference *p* < 0.001 from the STZ group after 33 days of the experiment (significant at *p* < 0.05, very significant p < 0.01, and highly significant *p* < 0.001)

LOAD group exhibited a significantly marked reduction in both total locomotor and exploratory activity (*p* < 0.001) in the open field. Crossed squares were decreased (5.70 ± 0.70 vs 38.20 ± 2.64) and number of times to cross the center area was inhibited (1.30 ± 0.15 vs 8.10 ± 0.56) when compared to the sham control group. Treatment with Donepezil or magnolol reversed these behavioral changes, showing increased crossed squares (24.10 ± 1.66 and 21.30 ± 2.72, *p* < 0.001) and center crossings (3.70 ± 0.30 and 4.50 ± 0.86, *p* < 0.001) compared to the LOAD group Fig. 5A and B. Nano-magnolol significantly improved the behavioral locomotor activity *p* < 0.001 showing a more pronounced anxiolytic and locomotor-enhancing effect in crossed squares (34.10 ± 1.56) and crossing center (10.40 ± 0.80). Also, only the Nano-treated group demonstrated a statistically significant enhancement *p* < 0.001 in grooming time unlike other treatments compared to AD group (27.67 ± 3.89 s) as shown in Fig. 5C.

### Novel Object Recognition (NOR)

The AD group demonstrated a significant decrease in the DI compared to the sham control group (*p* < 0.001), indicating impaired recognition memory (0.15 ± 0.003 vs 0.52 ± 0.04). All treatment groups including Donepezil, Magnolol and Nano-magnolol showed a significant increase in the DI compared to the LOAD group (*p* < 0.001), reflecting improvements in memory performance (0.64 ± 0.065, 0.55 ± 0.033 and 0.69 ± 0.043). The Nano-magnolol group showed the highest improvement among the treated groups, approaching values observed in the sham group as shown in Fig. 5D.

### Passive avoidance

**Fig. 6:**
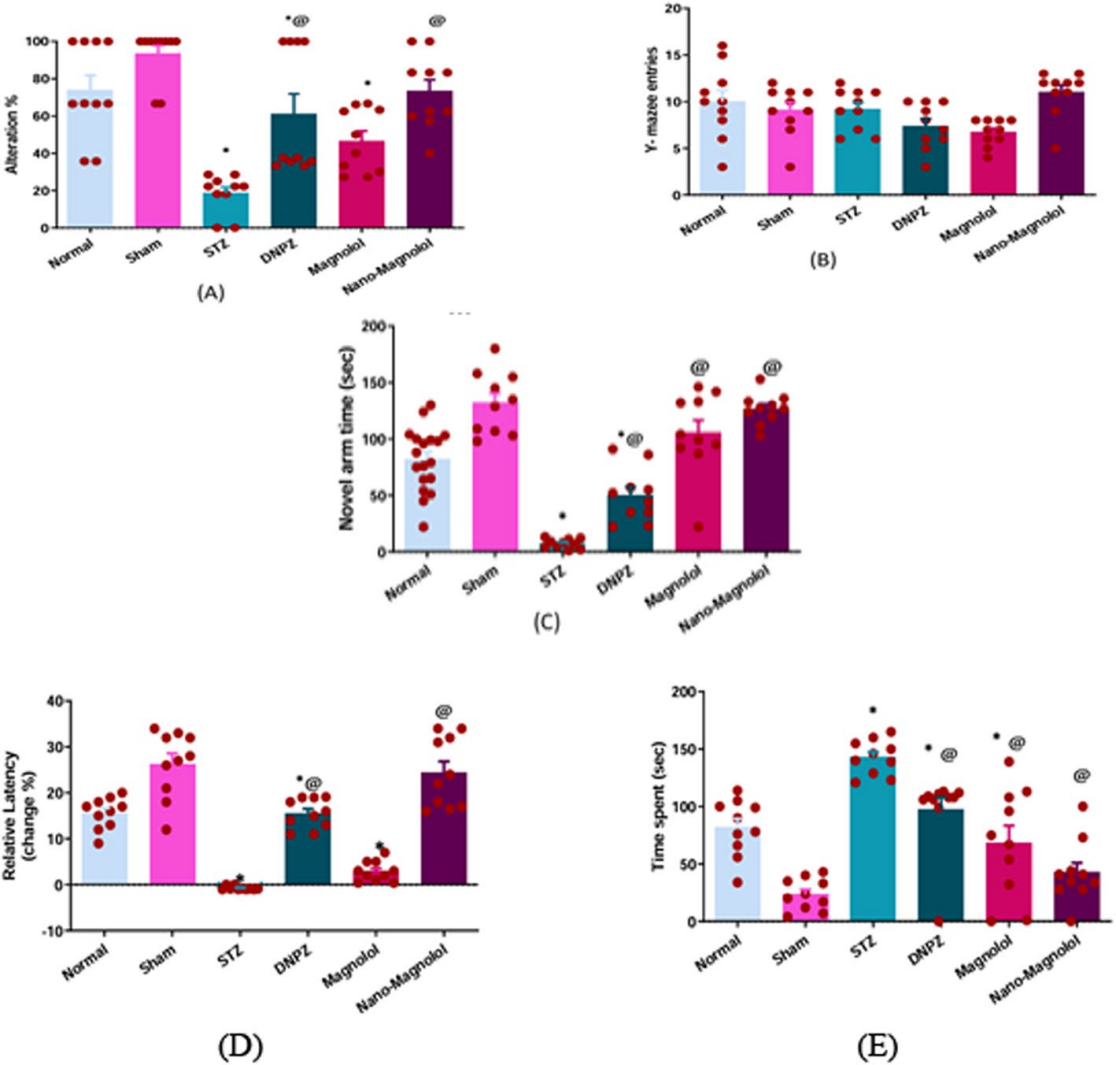
Y-maze **A**: alteration %, **B**: number of entries, **C**: novel arm time and Passive avoidance **D**: latency, **E**: time spent of all experimental groups. *Represents a significant difference *p* < 0.001 from the sham control group after 33 days of the experiment and @ represents a significant difference *p* < 0.001 from the STZ group after 33 days of the experiment (significant at *p* < 0.05, very significant *p* < 0.01, and highly significant *p* < 0.001)

LOAD group exhibited a significantly negative percent change in relative latency, reflecting memory decline compared to the sham control group (*p* < 0.001) (− 0.76 ± 0.13 *vs*. 26.30 ± 2.29). In contrast, all treatment groups showed positive percent change, Donepezil and Nano-magnolol groups exhibited significant improvements compared to the LOAD group (*p* < 0.001), (15.51 ± 1.02% and 24.45 ± 2.40%). The LOAD group spent noticeably more time in the dark compartment during the retention trial in contrast to the sham control group (*p* < 0.001) (142.80 ± 4.81 vs 23.50 ± 4.365 s). All treatment groups Donepzil, magnolol, and Nano-magnolol significantly reduced time spent in the dark compartment (*p* < 0.001) compared to the LOAD group (97.20 ± 10.87 s, 68.50 ± 14.95 s, and (42.40 ± 8.56 s) with Nano-magnolol demonstrating a significant improvement Fig. 6D and E.

### Y-maze test

The LOAD group had a severe impairment in all memory-related markers on the Y-Maze test. In contrast to the sham control group, working memory impairment was demonstrated by a considerably decreased spontaneous alternation percentage (*p* < 0.001), (18.52 ± 3.28% vs 93.33 ± 4.45%). All treated groups; i.e. Donepzil, magnolol, and Nano-magnolol showed a marked improvement as compared to the LOAD group (*p* < 0.001), (61.30 ± 10.54%, 46.66 ± 5.35%, and 73.23 ± 6.24%), The Nano-magnolol group showed the highest alternation percentage, nearing normal values as in Fig. 6A.The number of arm entries did not change significantly among groups as shown in Fig. 6B, indicating that the treatments did not effect on locomotor activity. The AD group spent considerably less time in the novel arm than the sham control group (*p* < 0.001), indicating spatial memory issues (7.20 ± 1.32 s vs 131.90 ± 8.72 s).Treatment groups Donepzil, magnolol, and Nano-magnolol, especially magnolol and Nano-magnolol, spent more time in the novel arm compared to AD (*p* < 0.001), demonstrating improved spatial recognition (50.0 ± 7.49), (105.20 ± 11.59), and (126.60 ± 4.29) as shown in Fig. 6C.

### Morris water maze test (MWM)

**Fig. 7:**
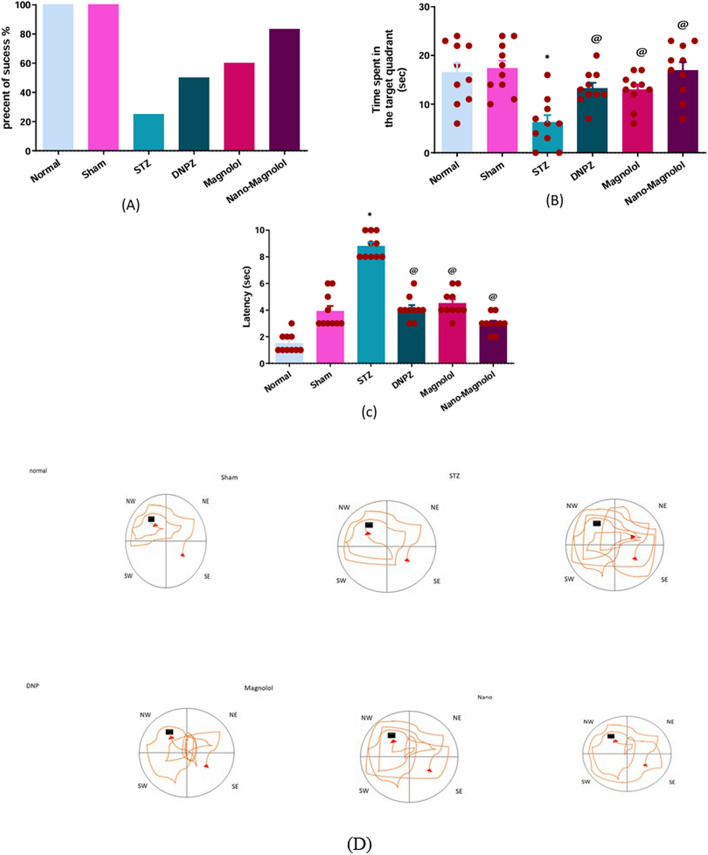
Morris water maze (MWM) **A**: percent of success, **B**: time spent in the target quadrant, **C**: latency, **D**: MWM tracking and path tracing patterns of all experimental groups. *Represents a significant difference *p* < 0.001 from the sham control group after 33 days of the experiment and @ represents a significant difference p < 0.001 from the STZ group after 33 days of the experiment (significant at *p* < 0.05, very significant *p* < 0.01, and highly significant *p* < 0.001)

In the Morris Water Maze test Fig. 7D, the LOAD group showed a significant decline in percent of success and duration of the target quadrant when compared to the sham control group (*p* < 0.001), (6.20 ± 1.56 s vs 17.30 ± 1.62 s). All treated groups Donepezil, magnolol, and Nano-magnolol showed varying degrees of significant improvement (*p* < 0.001), (13.30 ± 1.106 s), (12.90 ± 1.120 s), and (16.90 ± 1.74 s) as shown in Fig. 7A and B. Escape latency of the LOAD group showed a significant increase compared to the sham control group (*p* < 0.001), indicating impaired spatial learning and memory as shown in Fig. 7C. Notably, the Nano-magnolol group demonstrated a significant improvement in all parameters compared to the LOAD group, approaching normal control levels.

### Hippocampus AChE activity, GST, BACE1, and NF-KB

Biochemical analysis showed that, in the LOAD group there was a significant decline in GST activity and a marked elevation in AChE, NF-KB, and BACE1 levels when compared to the sham control group (*p* < 0.001) as shown in Table 1.

**Table 1:** The activity of AChE, levels of GST, BACE1, and NF-kB in hippocampus of all groups

Groups parameters	Normal	Sham	STZ	DNPZ	Magnolol	Nano-magnolol
AChE (U/mg protein)	1.03 ± 0.02	1.13 ± 0.02	2.03 ± 0.04^*^	1.10 ± 0.07^@^	0.93 ± 0.03^*@^	1.18 ± 0.04^@^
GST (µmol/min/mg protein)	5.445 ± 0.18	5.345 ± 0.06	2.113 ± 0.13*	3.450 ± 0.36*^@^	2.493 ± 0.13*	5.945 ± 0.38^@^
NF-KB (ng/mg protein)	3.05 ± 0.01	3.58 ± 0.05	5.28 ± 0.11*	3.97 ± 0.34^*@^	2.18 ± 0.17^*@^	3.66 ± 0.06^@^
Beta-secretase-1 (ng/mg protein)	3.125 ± 0.03	3.225 ± 0.07	8.650 ± 0.16*	4.986 ± 0.22^*@^	5.324 ± 0.08^*@^	3.61 ± 0.10^@^

The data (n = 10 rats) are presented as mean ± SEM. *Denotes a significant difference *p* < 0.001 from the sham control group, and @ denotes a significant difference *p* < 0.001 from the AD group, nuclear factor kappa B (NF-kB), glutathione-S-transferase (GST), and acetylcholinesterase (AChE) activity. One-way ANOVA was used for statistical analysis, and the Tukey multiple comparison post-hoc test was used thereafter (significant at *p* < 0.05, very significant *p* < 0.01, and highly significant *p* < 0.001)

**Fig. 8:**
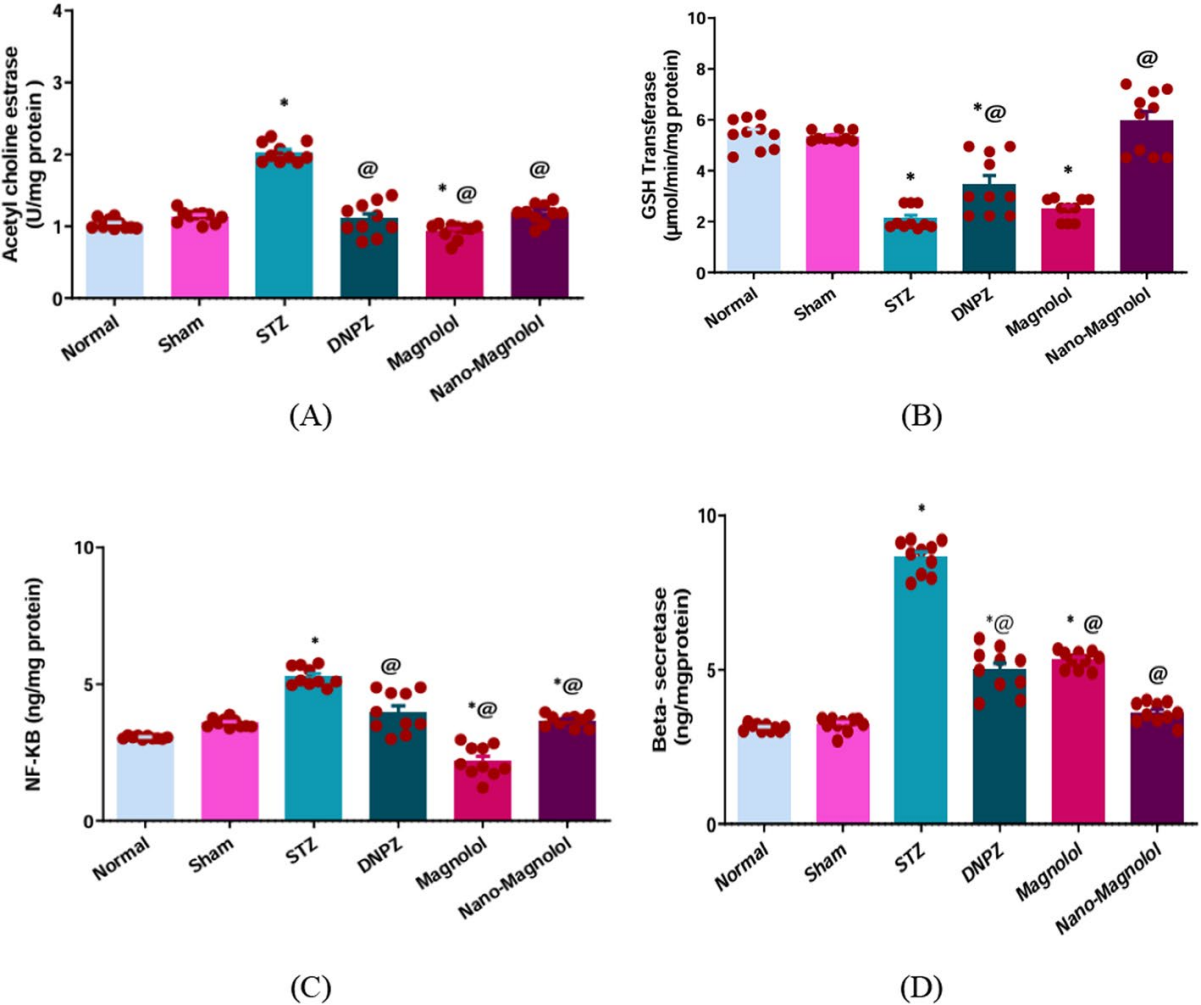
**A** AChE, **B** GST, **C** NF-kB, and **D** BACE1 levels in all experimental groups. *Represents a significant difference *p* < 0.001 from the sham control group after 33 days of the experiment and @ represents a significant difference *p* < 0.001 from the STZ group after 33 days of the experiment (significant at *p* < 0.05, very significant *p* < 0.01, and highly significant *p* < 0.001)

The most notable neuroprotective benefits were caused by Nano-magnolol therapy, which restored GST activity to nearly normal levels (*p* < 0.001) while considerably lowering AChE, NF-KB, and BACE1 levels. Additionally, all markers also produced significant, though comparatively moderate benefits with donepezil and magnolol as shown in Fig. 8.

#### Molecular docking study

BACE-1 active site has many important amino acids including (SER35, GLY230, PHE108, ASP32 and ASP228). In presence of water molecules, they participate in a conserved hydrogen bonding network that assists the catalytic reaction. Also, in the active BACE-1 structures, TYR71, as well as the neighboring residues of TRP76 and PHE108, ILE110 and TRP115 have key roles in the hydrophobic interactions formed to stabilize its reaction. TYR71 adopts a unique orientation to form hydrogen bonds in this structure. It seems that this network plays an important role in the catalytic reaction in BACE-1 [51].

By comparing the binding free energy of M7D (co-crystallized Pyrrolopiperazine BACE-1 Inhibitor) (− 8.908 kcal/mol) and magnolol (− 6.895 kcal/mol) it is clear that the studied compound (magnolol) has a very good affinity to the BACE-1 binding site residues.

Referring to the structure of magnolol which is characterized by the presence of phenolic and non-phenolic hydroxyl groups that act as hydrogen donors and acceptors in the formation of many hydrogen bonds with the side chains of the key amino acids such as (Tyr71, Ser35 and Asp32) as shown in Table 2 and supplementary Figs. S3–S5 strengthen the affinity of magnolol to the BACE-1 active site.

**Table 2:** The binding free energies (ΔG) and binding site residues of M7D and magnolol interactions with BACE-1

Ligands	ΔG (kcal/mol)	Interacted ligand binding residues
M7D	− 8.908	ASP228, THR231, LYS224, VAL332 and TYR71
Magnolol	− 6.895	PHE108, TYR71, SER35, ASP228, TYR198 and ASP32

## Discussion

The AD is recognized as the most cause of late-life dementia and a serious threat to public health [34, 57]. The ICV injection of STZ- induced an experimental model of sporadic AD or LOAD [25, 36] since it causes oxidative stress, a decreased cerebral energy metabolism, and neuro-inflammation processes that resulted in learning and memory deficits [60]. In current study, data showed that nano-magnolol restored the memory impairment and other pathological effects of STZ. The Nano formulation appears to have a greater therapeutic impact than conventional magnolol therapy. Nano formulation holds the possibility of new techniques to treat AD by allowing targeted delivery to specific brain regions, improved pharmaceutical stability and bioavailability, and the transfer of therapeutic molecules across the blood brain barrier. Numerous nano-systems have been developed to deliver drugs directly to brain cells, potentially boosting their efficacy with reducing their side effects [19, 32].

Comparing to control rats, the induced LOAD group showed a significant reduction in body weight, reflecting systemic deterioration and suspected metabolic dysregulation, which are frequently linked to STZ-ICV-induced neurotoxicity. It was suggested that, STZ-LOAD induction resulted in enough impairment to reduce food intake and make weight gaining more difficult [36, 65]. On the other hand, the treated groups (Donepezil, magnolol, and Nano-magnolol) maintained noticeably greater weight gain percentages compared with untreated. The increased in body weight could be a result of treatments’ better overall health and decreased systemic toxicity.

Behavioral examination revealed that, the EPM test demonstrated considerable anxiety-like behavior in the LOAD group, as evidenced by decreased time spent and fewer admissions into the open arms. This behavior is consistent with elevated avoidance and emotional reactivity seen in animal models of AD, where hippocampus damage affects emotional regulation and exploratory drive [12, 84]. Nano-magnolol administration improved open arm activity, suggesting an anxiolytic impact. Donepezil and magnolol treatment also improved open-arm exploration but less than nano-magnolol, demonstrating its role in regulating anxiety and emotional equilibrium, compatible with previously observed anxiogenic effects in both mouse and rat models [25].

The Open Field Test indicated that LOAD inhibited exploratory activity and locomotion, reflected by a decreased number of crossings and center entries, with elevated anxiety. Nano-magnolol treatment improved locomotor behavior, indicating that it has anxiolytic and cognitive-enhancing characteristics. Donepezil and magnolol both markedly improved activity of rats but less than nano-magnolol. This is in agreement with our results, donepzil and magnolol had ameliorative activity in Alzheimer-like experimental animal model [80].

In the current study, LOAD group had a substantial decrease in discrimination index during the Novel Object Recognition test, demonstrating impairment in recognition memory and object discrimination in accordance to previous reports [39]. These findings are consistent with the known impairment of hippocampus in AD [26]. Treatment with nano-magnolol, magnolol, and Donepezil considerably recovered recognition performance, indicating improvements in visual-spatial and short-term memory.

The Passive Avoidance test demonstrated that the Alzheimer group exhibited lower step-through latency, alongside more time spent in the dark compartment, indicating diminished long-term memory and fear-associated learning [39].

Treatments with Nano-magnolol, magnolol, and donepezil enhanced avoidance behavior, implying that unpleasant memory consolidation had been restored. The LOAD (STZ‑ICV) induction showed a substantial reduction in spontaneous alternation percentage and a shorter time spent in the novel arm than the normal and sham control groups, indicating a profound impairment in working and spatial memory in the Y-Maze test. This is consistent with findings from other STZ-ICV studies that reveal similar cognitive decline, indicating deficits in hippocampus-dependent memory tasks without alterations in general locomotor activity [59]. The restoration of working and spatial memory was demonstrated by the considerable recovery of both alternation rate and new arm exploration following treatment with nano-magnolol. Its effectiveness was as comparable to or in some cases better than, that of donepezil and magnolol, highlighting the potential neuroprotective and synaptic-plasticity-enhancing properties of nano-magnolol.

Interestingly, there were no noticeable differences in the overall number of arm entries between all groups, indicating that variations in movement did not interfere with memory restoration. These results are consistent with other studies in STZ-ICV models showing that treatment effects on memory, not general activity, are responsible for cognitive improvements [43].

Significant spatial learning deficits were observed in the AD (STZ‑ICV) group in the MWM test indicating impaired hippocampal-dependent reference memory. These results are in line with earlier study showing comparable outcomes in animals after AD [85]. In comparison to the LOAD group, treatment with nano-magnolol increased escape latency, percent success, and time spent in the target quadrant, indicating effective memory and spatial learning restoration. Similar patterns on other treatments were documented by Noor et al. [50], who reported that curcumin nanoparticles corrected deficits caused by STZ-ICV in water maze.

In current study, the AChE activity was much higher in the LOAD group than in the sham control group, reflecting cholinergic dysfunction as a crucial feature of LOAD. This result is consistent with earlier study showing an increase in AChE after ICV- STZ injection [61]. Donepezil’s function as a cholinesterase inhibitor was confirmed by the considerable reduction in enzyme activity following treatment [80]. Interestingly, Nano-magnolol also significantly reduced AChE levels in comparison to ICV-STZ group confirming its modulatory effect on cholinergic transmission. These findings demonstrated the potential of nano-magnolol to improve biochemical and cognitive outcomes and emphasize the therapeutic significance of addressing cholinergic dysfunction in LOAD model.

The development of cognitive decline associated with LOAD is linked to elevated levels of Aβ aggregation in the brain, which in turn are mediated by increased BACE1 expression [6, 56]. The BACE1 is a necessary enzyme for proteolytic cleavage of the amyloid precursor protein to produce the naturally occurring Aβ peptides, which contribute to AD pathogenesis. In the current study, the STZ-ICV-induced LOAD group had significantly higher BACE1 protein than the sham control group, supporting the concept that brain insulin resistance and metabolic dysfunction can worsen amyloidogenesis. Previous research has shown that oxidative stress and neuro-inflammation, which are common in STZ models, can activate BACE1 expression via the NF-KB signaling pathway [9, 27, 56]. Treatment with nano-magnolol significantly reduced BACE1 levels, most likely because it could control inflammatory, oxidative cascades, and amyloidogenesis. Additionally, molecular docking research demonstrated that magnolol has a high binding affinity to the BACE1 catalytic site, indicating the possibility of direct enzymatic inhibition and supporting its function in reducing the production of Aβ. Given its multi-target neuroprotective potential, nano-magnolol showed a more marked decrease in BACE1 than donepezil, which had a great impact. The molecular docking computational findings supported the in vivo findings that nano magnolol markedly decreased BACE1 levels. A direct inhibitory mechanism may be responsible for nano-magnolol’s anti-amyloidogenic and neuroprotective actions in the (STZ-ICV) AD model, as evidenced by its significant in silico affinity for the BACE1 catalytic site.

Furthermore, increased Aβ synthesis affects BACE1 promoter transactivation via an NF-KB dependent mechanism. Active NF-kβ can influence AD pathogenesis by increasing neuro-inflammatory cytokine production, as evidenced by activated microglia and astrocytes [46]. This process is typically associated with an increase in reactive oxygen species (ROS) and oxidative stress (OS). The brain’s susceptibility to OS is regarded as a critical risk factor in AD. Aβ promotes OS, which increases Aβ deposition [73].

In contrast to sham controls in our study, the AD group showed noticeably elevated NF-κB levels, revealing deep neuro-inflammatory changes caused by STZ injection. This is in aligns with the proven role of NF-*K*B as a master regulator of inflammatory signaling in AD [69]. As its anti-inflammatory properties, nano-magnolol treatment dramatically reduced NF-Kβ in hippocampus tissue, demonstrating the importance of inflammation modulation for cognitive recovery. As NF-κB activation triggers neuro-inflammation, enhanced BACE1 promotes amyloid-beta production. The observed reduction in both markers in treated groups specifically nano-magnolol supports a potential anti-inflammatory and anti amyloidogenic effect, which is consistent with recent research [66, 87].

Data obtained indicted that, the activity of GST was significantly lowered in the LOAD group compared with sham group, indicating a diminished ability to detoxify antioxidants under oxidative stress. Studies in AD models have demonstrated higher antioxidant defense after magnolol therapy [83], which supports the discovery that nano-magnolol dramatically restored GST activity, indicating enhanced glutathione-mediated detoxification. These results demonstrate the importance of strengthening the endogenous antioxidant system to reduce oxidative damage associated with AD.

## Conclusion

It was concluded that, Nano-magnolol showed a potential effective in neurodegenerative disease (LOAD) via behavioral and cellular pathways as cholinergic function, amyloidogenesis, neuroinflammation, and oxidative stress reflecting its potent neuroprotective efficacy in AD treatment.

## Limitations

These findings should be validated in bigger and more diversified animal models, encompassing both sexes and age groups. It is advised that the studied chemicals’ long-term safety and effectiveness be looked into. Further molecular investigations may provide additional details on the mechanisms that underlie disease. The relative efficacy may be clarified by comparison studies with other conventional treatments. To determine clinical relevance, translational studies in human subjects are required.

## Supplementary Information

Additional file1 (DOCX 806 kb)

## Data Availability

All datasets generated or analyzed during this study are included in the manuscript.

## List of abbreviations

Ache: Acetylcholinesterase
AD: Alzheimer’s disease
APP: Amyloid precursor protein
Aβ: Β-Amyloid peptide
BACE1: B-secretase1
DI: Discrimination index
EOAD: Early-onset AD
EPM: Elevated plus maze
GST: Glutathione-S-transferase
ICV: Intracerebroventricular
LOAD: Late-onset Alzheimer’s disease
MWM: Morris water maze
NOR: Novel object recognition
NF-Kb: Nuclear factor kappa B
OFT: Open field test
OS: Oxidative stress
PA: Passive avoidance
PBS: Phosphate buffer saline
PVA: Polyvinyl alcohol
ROS: Reactive oxygen species
STZ: Streptozotocin
TEM: Transmission electron microscopy
