# Supplementary material for: Nano-magnolol enhances the modulatory effects of magnolol on cognitive performance and BACE1-related biochemical changes in an STZ-induced rat model of Alzheimer’s disease
Source: Discov Nano. 2026 Apr 9;21(1):110. doi: 10.1186/s11671-026-04506-9 (PMC13065978; doi:10.1186/s11671-026-04506-9)
Supplement: Supplementary file 1 — Additional file1 (DOCX 806 kb) [file 11671_2026_4506_MOESM1_ESM.docx]

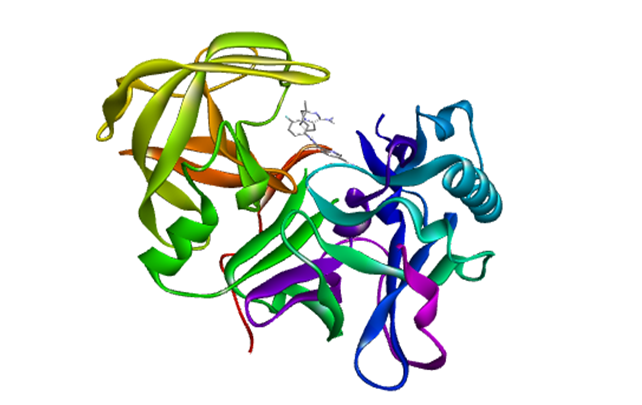


**S1: 3D structure of BACE1 complexed with M7D inhibitor**

| 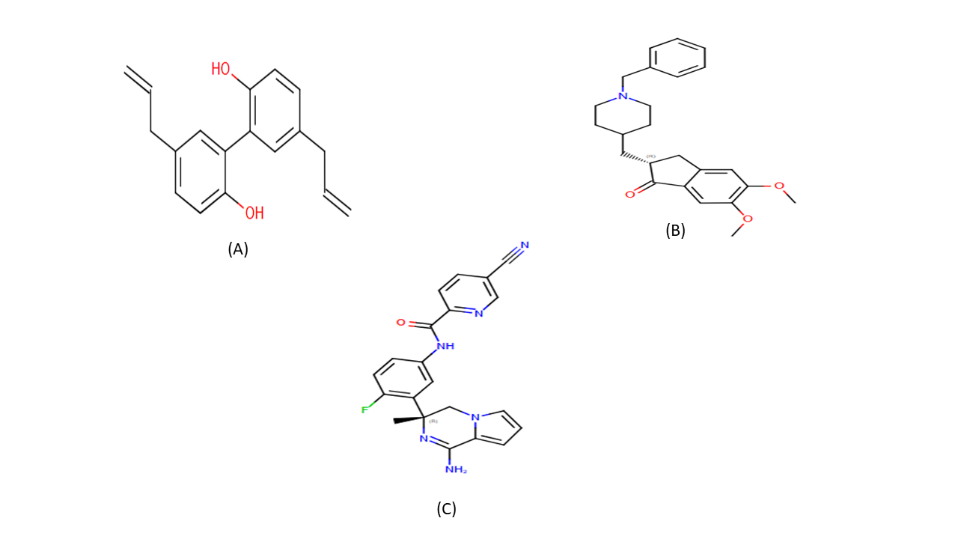 |
| --- |
| 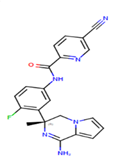 |
| (B) |

**S2: Ligands were used in molecular docking a) Magnolol, and b) M7D co-crystallized ligand**


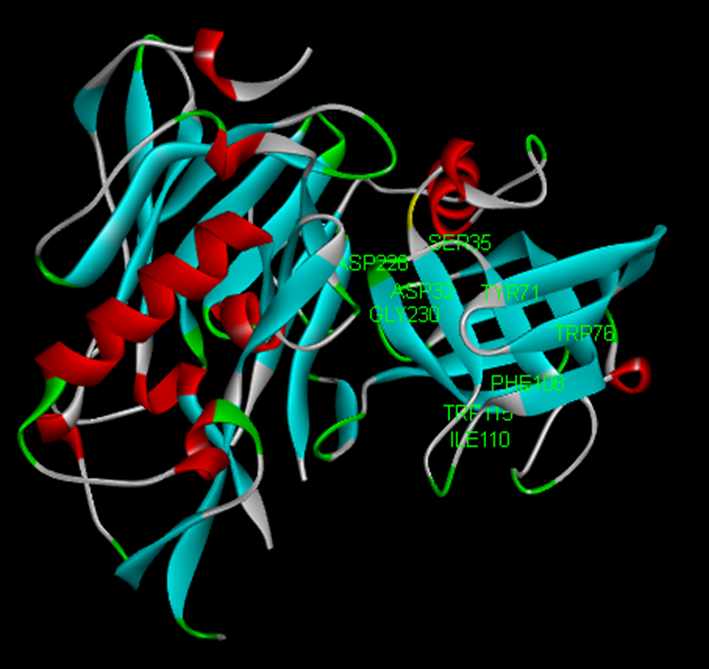


**S3: BACE1 3D structure referring to the important amino acids in its active site**

| **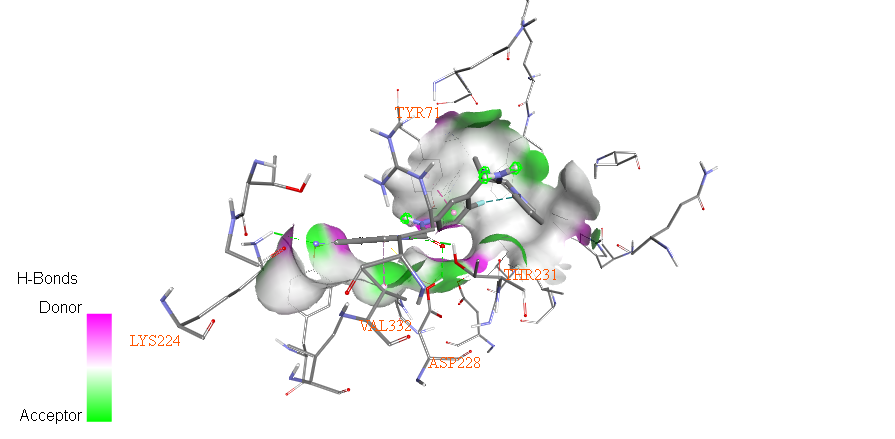** |
| --- |
| 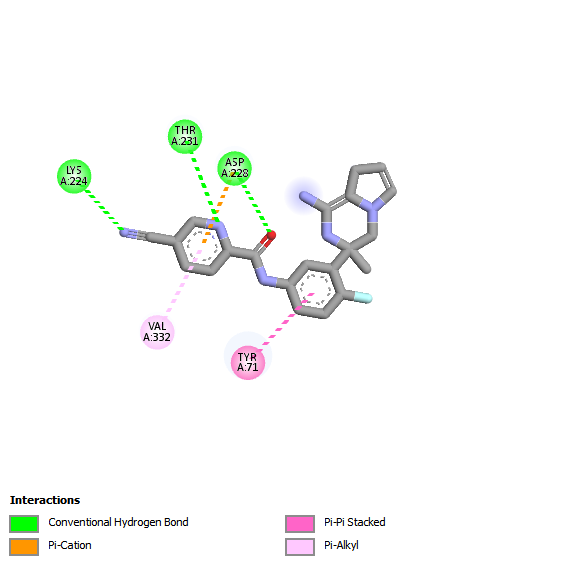  **S4: Interactions of BACE-1 residues with co-crystalized inhibitor M7D; 3D and 2D figures respectively** |

|  |
| --- |
|  |

| 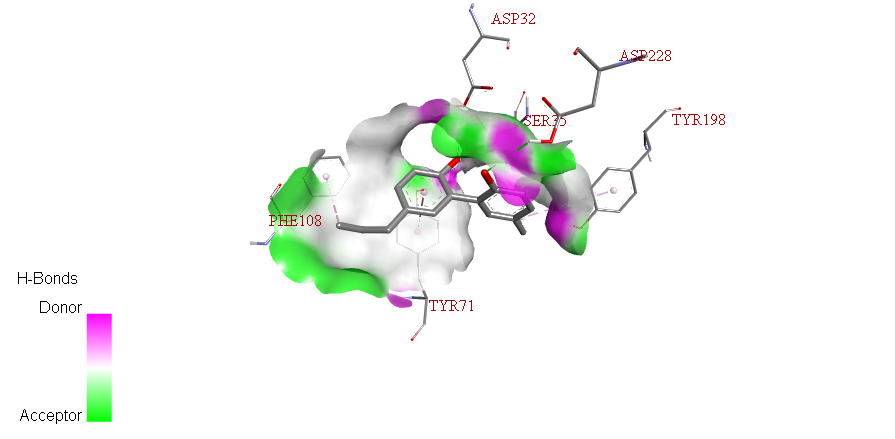 |
| --- |
| 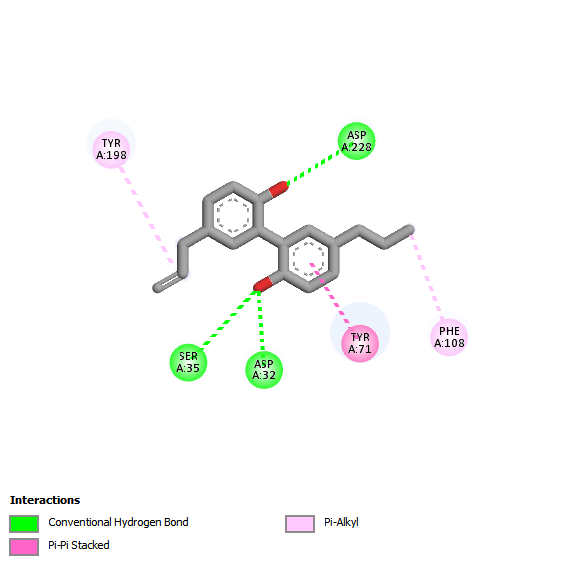  **S5: Interactions of BACE-1 residues with magnolol; 3D and 2D figures respectively** |
